# Biogenesis and function of circular RNAs and their implications in the Down syndrome brain

**DOI:** 10.3389/fgene.2025.1708015

**Published:** 2025-11-27

**Authors:** Shahidee Zainal Abidin, Nurul Nadirah Razali, Pike-See Cheah, King-Hwa Ling

**Affiliations:** 1 Faculty of Science and Marine Environment, Universiti Malaysia Terengganu, Terengganu, Malaysia; 2 Research Interest Group Biological Security and Sustainability (BIOSES), Faculty of Science and Marine Environment, Universiti Malaysia Terengganu, Terengganu, Malaysia; 3 Department of Human Anatomy, Faculty of Medicine and Health Sciences, Universiti Putra Malaysia, Serdang, Selangor, Malaysia; 4 Malaysian Research Institute on Ageing (MyAgeing®), Universiti Putra Malaysia, Serdang, Selangor, Malaysia; 5 Department of Biomedical Sciences, Faculty of Medicine and Health Sciences, Universiti Putra Malaysia, Serdang, Selangor, Malaysia

**Keywords:** circular RNA, Down syndrome, miRNA sponging, cognitive development, gene regulation

## Abstract

Circular RNAs (circRNAs), a class of covalently closed, non-coding RNAs, have recently emerged as crucial regulators of gene expression. They exert their roles through microRNA (miRNA) sponging, transcriptional regulation, and interactions with RNA-binding proteins (RBPs). Increasing evidence suggests that circRNAs play important roles in neurodevelopmental disorders, including Down syndrome (DS). DS is a condition caused by trisomy of chromosome 21 and characterised by intellectual disability (ID), neuroinflammation, and increased risk of early-onset Alzheimer’s disease (AD). Aberrant circRNA expression in DS may contribute to pathogenesis by disrupting competing endogenous RNA (ceRNA) networks, modulating synaptic plasticity, and influencing key molecular pathways, including EZH2-mediated chromatin remodelling, immune response regulation, and neuronal differentiation. Despite these emerging insights, the role of circRNAs in DS remains largely underexplored compared to their well-recognised functions in cancer and other neurological disorders. Most current studies have focused on transcriptomic analyses, identifying differentially expressed circRNAs and predicting their interactions with miRNAs and mRNAs. However, these findings require further experimental validation to uncover the precise mechanisms through which circRNAs contribute to DS pathophysiology. This review highlights the association of circRNAs with DS, emphasising their dysregulation and mechanistic interactions with miRNAs and mRNAs. It further explores how these circRNA-mediated mechanisms may contribute to intellectual disability and impaired neurodevelopment, based on current evidence.

## 1 Introduction

Down syndrome (DS) is a genetic disorder caused by the aneuploidy of human chromosome 21 (Hsa21). According to the World Health Organisation (WHO), the incidence rate of DS is between 1 in 1,000 to 1 in 1,100 live births worldwide. The incidence rate of Hsa21 non-disjunction increases with advanced maternal age (Allen et al., 2009). The DS is classified into three types: (1) trisomy 21, (2) translocation DS, and (3) mosaic DS (Plaiasu, 2017). Trisomy 21 is the most common form of DS, affecting approximately 95% of people with DS. This form of DS is due to the failure of the homologous Hsa21 to segregate during meiosis, leading to an extra Hsa21. The occurrence of translocation DS is infrequent, in which one of the Hsa21 is translocated to another chromosome. The mosaicism in DS is rare, affecting 2%–3% of the DS population. In this condition, some cells contain three copies of Hsa21, while others exhibit typical disomy of chromosome 21 (Plaiasu, 2017).

In 1959, Lejeune Gautier and Turpin reported that trisomy 21 was the genetic cause of DS (Bouizegarène et al., 2008). Since then, the identification of pathogenic mechanisms through which trisomy 21 induces the clinical phenotype has been explored. The most prominent hallmark of the DS phenotype is neurodevelopmental abnormalities. This has been determined by delay or failure in motor skills, speaking and reading with short-term memory impairment, and learning difficulties (Malak et al., 2015; Wilson et al., 2019). The conditions are due to impairment of brain development, which subsequently led to abnormalities in brain structure and function (Dierssen, 2012; Vacca et al., 2019). These developmental disruptions manifest in multiple ways throughout the brain, giving rise to distinct structural and functional changes that underlie the neurological and cognitive features of the disorder. Collectively, these changes underlie the intellectual disability (ID) and cognitive decline observed in individuals with DS.

Structurally, individuals with DS exhibit an overall smaller brain size, with notable reductions in the frontal and temporal lobes, hippocampus, and cerebellum (Adamo et al., 2025). The hippocampus, which is essential for learning and memory, is underdeveloped and exhibits impaired synaptic plasticity (Robinson et al., 2024). The cerebellum shows a decrease in granule cells, leading to difficulties in motor coordination and learning (Feely et al., 2025). Additionally, the cerebral cortex is thinner and presents a simpler folding pattern, accompanied by abnormal dendritic spines that diminish neuronal connectivity (Robinson et al., 2024). Functionally, the DS brain exhibits reduced neurogenesis, delayed myelination, and an imbalance between excitatory (glutamate) and inhibitory (GABA) signalling, along with deficits in cholinergic, serotonergic, and dopaminergic systems (Robinson et al., 2024).

Furthermore, the aetiology of the DS phenotype is complex and includes other mechanisms. In recent years, non-coding RNAs such as microRNAs (miRNAs) and circular RNAs (circRNAs) have been increasingly implicated in DS. Their involvement appears to be particularly relevant to the ID associated with the condition (Brás et al., 2017; Karaca et al., 2017; Zbucka-Kretowska et al., 2019). Advanced maternal age increases trisomy 21 risk via meiotic errors, causing global transcriptional imbalance and circRNA dysregulation. Additionally, oocyte ageing and epigenetic changes may further modulate trisomy-related circRNA expression. The circRNA is characterised by a unique back-splicing event, in which the downstream 5′ splice site of an exon covalently joins to the upstream 3′ splice site. The back-splicing event can happen either within the same exon or with another exon. As a result, circRNA escapes canonical cap-dependent translation as it lacks the 5′ cap necessary for the docking of ribosomes. This mechanism demonstrated circRNA regulation, refuting the initial misbelief that circRNAs are anomalies or transcriptional noise arising from erroneous RNA splicing (Ragan et al., 2019; Xiao et al., 2020). Numerous studies have reported high circRNA enrichment in the brain, which is potentially due to the accumulation of cells with low division rates, such as neurons. Nevertheless, the circRNA expression profile across different developmental brain stages, particularly in DS, remains limited. Therefore, this review aims to highlight the association of circRNAs with DS, focusing on their dysregulation, interactions with miRNAs and mRNAs, and the potential mechanisms by which circRNAs may contribute to ID and impaired neurodevelopment.

## 2 Overview of circRNAs

The circRNA is a member of the non-coding RNA family produced by a non-canonical splicing event known as back-splicing (Kristensen et al., 2017). During back-splicing, a downstream splice donor site is covalently linked to an upstream splice donor site. In recent years, high-throughput RNA sequencing (RNA-seq) and circRNA-specific bioinformatics algorithms have identified thousands of circRNAs in eukaryotes, fungi, protists, plants, worms, fishes, insects and mammals (Ivanov et al., 2014; Jeck et al., 2012; Salzman et al., 2013; Wang et al., 2014; Westholm et al., 2014). Interestingly, the circRNA is also found to have a tissue-specific expression (Salzman et al., 2013; Maass et al., 2017; Xia et al., 2016). Most circRNAs are expressed from known protein-coding genes and consist of a single exon or multiple exons (Guo et al., 2014). Intriguingly, many circRNAs are upregulated during neurogenesis, and some are enriched in synapses (Hanan et al., 2016). The circRNA has been observed to be actively produced by terminally differentiated cells, particularly in the nervous system, such as neurons (Van Rossum et al., 2016).

### 2.1 Biogenesis of circRNA

**FIGURE 1:**
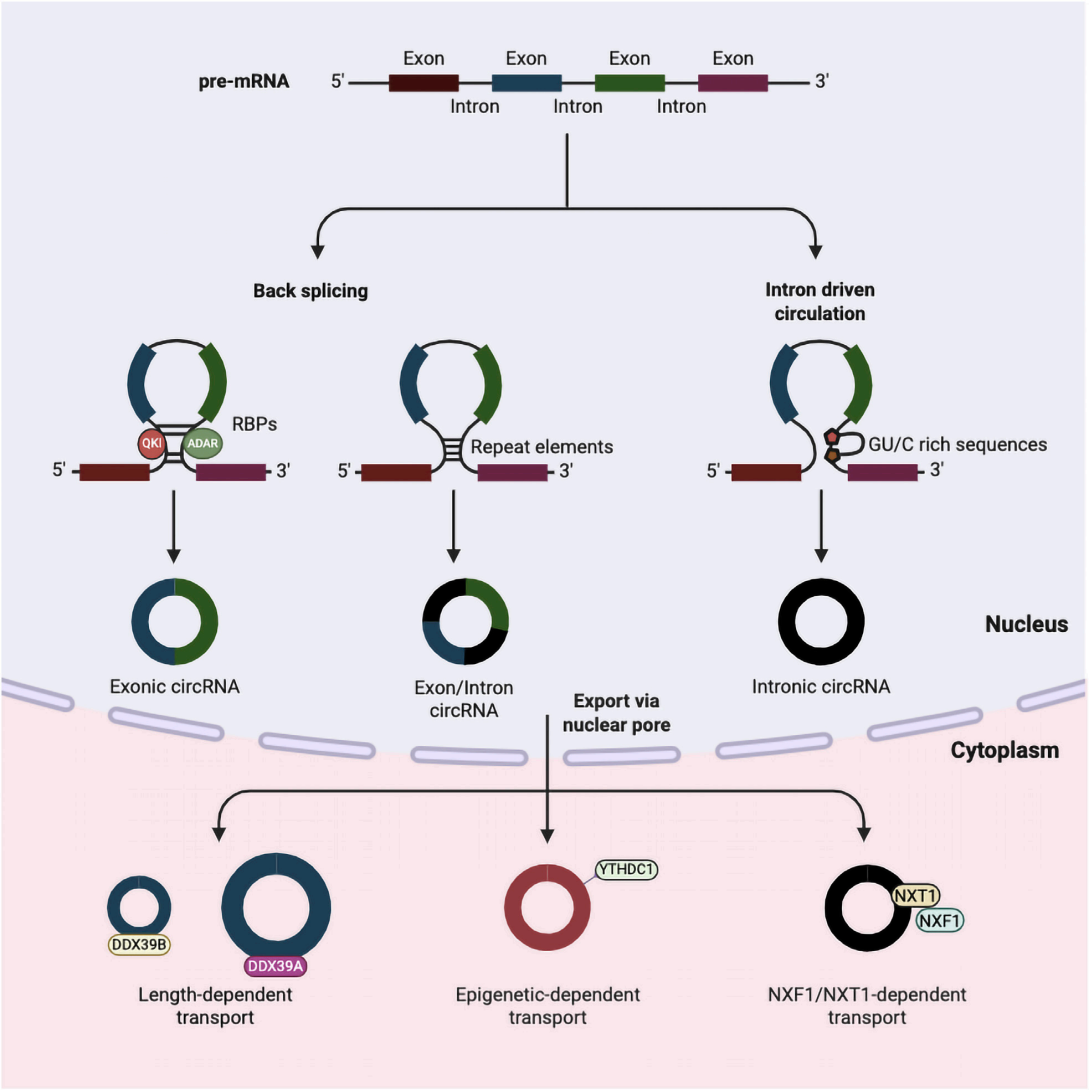
Biogenesis and nuclear export of circRNAs. Pre-mRNA undergoes back-splicing or intron-driven circularisation to generate three main types of circRNAs: exonic circRNAs, exon–intron circRNAs, and intronic circRNAs. Exonic circRNAs are formed by RNA-binding proteins (RBPs), such as QKI and ADAR; exon–intron circRNAs are facilitated by repeat elements; and intronic circRNAs arise from intron-driven circulation involving GU/C-rich sequences. Following formation, circRNAs are exported from the nucleus to the cytoplasm through distinct pathways, including length-dependent transport (DDX39B/DDX39A), epigenetic-dependent transport (YTHDC1), and NXF1/NXT1-dependent transport. Created in BioRender. Ling, K. (2025) https://BioRender.com/etqc9ej.

CircRNAs are produced from precursor mRNAs by RNA polymerase II. This occurs through back-splicing, where the 3′ splice site connects to an upstream 5′ splice site, forming a closed loop (Chen, 2020). There are three main models of circRNA formation: (1) intron pairing–driven, (2) RNA-binding protein (RBP)–driven, and (3) lariat-driven circularisation (Figure 1) (Aufiero et al., 2019). Based on their compositions and circularisation mechanism, circRNAs are classified into three types: (1) exonic circRNAs (ecircRNAs), (2) circular intronic RNAs (ciRNAs), and (3) exon–intron circRNAs (EIciRNAs) (Rybak-Wolf et al., 2015; Li Z. et al., 2015). EcircRNAs are primarily generated from one or more exons of pre-mRNA (Meng et al., 2016), whereas ciRNAs originate from introns through spliceosome-mediated splicing (Du et al., 2016a). EIciRNAs, on the other hand, contain both exonic and intronic sequences (Guo et al., 2014).

In circRNA biogenesis, RBPs facilitate circularisation, while adenosine deaminase acting on RNA (ADARs) contribute to circRNA formation via A-to-I editing, guided by reverse complementary sequences. ADARs influence the stability of RNA secondary structures. This modulation affects the binding of regulatory RBPs, thereby either suppressing or facilitating back-splicing events (Ivanov et al., 2014; Shen et al., 2022). In addition, the spliceosome has been implicated in circRNA formation in a manner analogous to canonical splicing, although the underlying mechanisms remain incompletely understood (Ashwal-Fluss et al., 2014; Chen and Yang, 2015; Awasthi et al., 2018). Within the intron lariat–driven circularisation, exon skipping generates an mRNA lacking specific exons, while the associated lariat structure has the potential to create a circRNA once it escapes debranching. Sequence elements, such as a 7-nucleotide GU motif and an 11-nucleotide C-rich motif, located near the 5′ splice sites, protect lariat structures from debranching by enzymes like DBR1, thereby promoting circularisation (Zhang et al., 2013; Pisignano et al., 2023).

CircRNAs are also subject to post-transcriptional regulation through various RNA modifications, among which N6-methyladenosine (m^6^A) represents the most abundant internal modification in eukaryotic cells. For example, m^6^A-modified exons positioned near the start and stop widely codons of mRNAs can undergo back-splicing in a process mediated by the nuclear m6A reader protein YTHDC1 (Dattilo et al., 2023; Di Timoteo et al., 2020; Tang et al., 2020).

### 2.2 Transportation and localisation

During biogenesis, circRNAs are transported into the cytoplasm in a size-dependent manner, involving RBPs, export receptors, and RNA helicases (Jeck et al., 2012; Guo et al., 2014; Salzman et al., 2012). The major pathway for circRNA nuclear export depends on Ran-GTP–binding export receptors (Chen et al., 2022; Ngo et al., 2024), which mediate transport through the nuclear pore complex. In mammals, exportin-2 (XPO2) mediates the export of nearly 80% of the most abundantly expressed circRNAs (Ngo et al., 2024). Another receptor, exportin-4 (XPO4), facilitates the transport of a distinct group of exonic circRNAs that are highly expressed in brain tissue (Chen et al., 2022). In addition to Ran-GTP–dependent exportins, ATP-dependent RNA helicase DDX39A (also known as nuclear RNA helicase URH49) and spliceosome RNA helicase DDX39B (also known as UAP56) play critical roles in this process.

URH49 primarily mediates the export of short circRNAs (<400 nucleotides), whereas UAP56 facilitates the export of longer circRNAs (>1,200 nucleotides) (Huang et al., 2018). Other studies suggest slightly different length thresholds: DDX39A promotes the export of circRNAs shorter than 700 nt, and DDX39B handles those longer than 800 nt, suggesting that size-dependent export requirements may vary across species (Huang et al., 2018; Li et al., 2018). This length-dependent export mechanism appears to be evolutionarily conserved. Notably, canonical mRNA export factors such as NXF1 (Nuclear Export Factor 1), ALYREF (ALY/REF Export Factor), and GANP (Germinal Center–Associated Nuclear Protein) contribute only minimally to circRNA transport (Ngo et al., 2024). Nonetheless, the NXF1–NXT1 pathway, typically involved in mRNA export, has been implicated in the transport of GC-rich ciRNAs, underscoring the mechanistic complexity and diversity underlying circRNA export (Wang et al., 2021).

RNA modifications, particularly m^6^A, play a crucial role in regulating the stability and subcellular localisation of circRNA. The nuclear m^6^A reader protein YTHDC1 facilitates the export of specific m^6^A-modified circRNAs, such as circNSUN2 and circRNA3634 (Chen et al., 2019; Song et al., 2023). However, this pathway is not universally required for all m^6^A-modified circRNAs. For example, although YTHDC1 knockdown reduces the biogenesis of circ-ZNF609, its nuclear export and stability remain unaffected (Di Timoteo et al., 2020). Beyond intracellular regulation, circRNAs have also been identified in exosomes (Bao et al., 2015), extracellular vesicles derived from intraluminal vesicles within multivesicular bodies. Exosomes serve as key mediators of intercellular communication by transporting diverse biomolecules, including RNAs, proteins, and lipids (Dou et al., 2016; Van Niel et al., 2018). Intriguingly, circRNAs are often enriched in exosomes relative to their levels in parental cells, indicating selective sorting mechanisms (Li Y. et al., 2015). Despite these observations, the molecular determinants governing circRNA packaging into exosomes remain poorly understood, representing an important area for future investigation.

### 2.3 Stability and degradation

**FIGURE 2:**
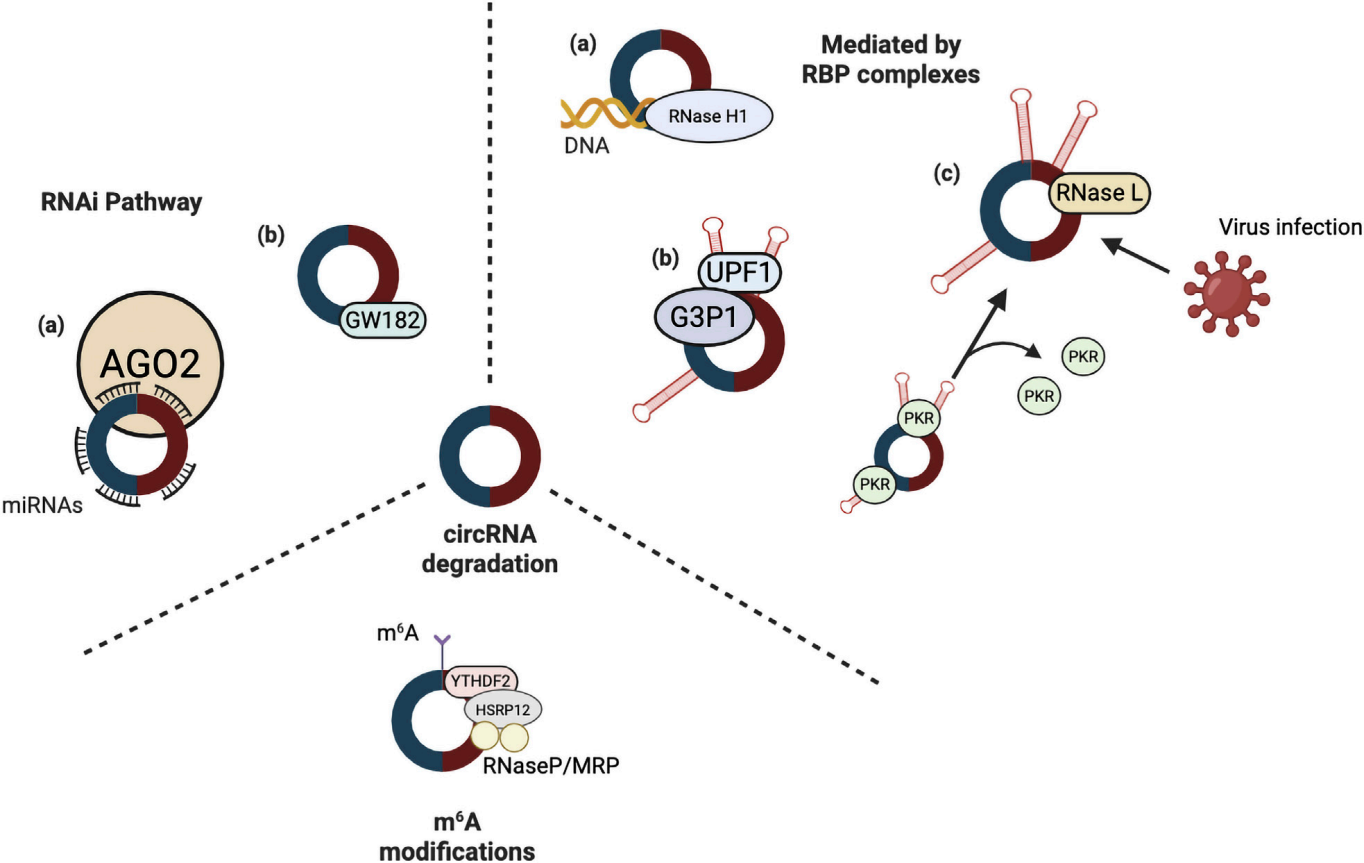
Pathways involved in circRNA degradation. Several cellular mechanisms tightly regulate CircRNA stability. (i) RNAi pathway: AGO2, in association with miRNAs, can guide the degradation of circRNAs, often mediated through GW182, linking circRNAs to post-transcriptional silencing complexes. (ii) m^6^A modification–dependent degradation: m^6^A marks on circRNAs recruit the m^6^A reader YTHDF2, which interacts with HSRP12 and facilitates the recruitment of RNase P/MRP endoribonucleases, thereby promoting selective circRNA decay. (iii) RBP complex–mediated degradation: Several RBP complexes participate in circRNA turnover. For instance, RNase H1 can degrade circRNAs that form DNA–RNA hybrids; UPF1, together with G3BP1, recognises specific circRNA structures to promote their clearance; and RNase L is activated during viral infection, leading to circRNA cleavage and the subsequent release of PKR, which enhances antiviral responses. Created in BioRender. Ling, K. (2025) https://BioRender.com/q5kq0pa.

CircRNAs are covalently closed-loop structures that lack free 5′ and 3′ ends, a feature that confers greater stability and resistance to exonuclease-mediated degradation compared to linear RNAs (Zhang et al., 2016). Consequently, their turnover requires mechanisms distinct from those governing linear RNAs. Several factors have been implicated in regulating circRNA stability and degradation dynamics, including endonuclease activity, miRNA binding, secondary structure formation, RNA–DNA hybridisation, and m^6^A modification (Figure 2). In endonuclease-mediated pathways, circRNA degradation often involves RNA interference (RNAi). This process requires Argonaute-2 (AGO2) endonuclease, guided by miRNAs. For instance, in mice, *miR-1224* binds to the precursor of circRNA-filip1l and promotes its degradation in an AGO2-dependent manner (Pan et al., 2019). Likewise, *miR-671* directs AGO2-mediated cleavage of circLINC00632 (also known as circCDR1as) (Hansen et al., 2011; Piwecka et al., 2017). Additionally, TNRC6A (also known as GW182), a core component of the RNAi machinery, has been implicated in circRNA decay through mechanisms independent of canonical RNAi pathways (Jia et al., 2019).

Moreover, circRNAs are also susceptible to degradation mediated by RBP complexes. For example, the RNA helicase UPF1 can unwind circRNA secondary structures, thereby enabling subsequent cleavage by the endonuclease G3BP1 (G3BP Stress Granule Assembly Factor 1) (Fischer et al., 2020). In some cases, circRNAs such as ci-ankrd52 form stable RNA–DNA hybrids at their transcription sites, giving rise to R-loops that are specifically recognised and degraded by RNase H1 (Li X. et al., 2021). During viral infection, circRNAs forming short RNA duplexes of 16–26 bp are targeted by RNase L, an endonuclease that plays a critical role in antiviral defence. This degradation facilitates the activation of PKR, a double-stranded RNA–dependent protein kinase, which in turn suppresses both viral and host protein synthesis (Burke et al., 2021; Liu et al., 2019; Zheng, 2019).

The presence of m^6^A modifications can increase the susceptibility of circRNAs to endonucleolytic cleavage. Specifically, m^6^A-marked circRNAs are recognised and targeted for degradation through the coordinated action of heat-responsive protein 12 (HRSP12) and the ribonuclease P/multidrug resistance-associated protein 1 (RNase P/MRP) complex (Park et al., 2019). This process is mediated by the m^6^A reader protein YTHDF2, which interacts with HRSP12, thereby facilitating the recruitment of the RNase P/MRP complex and promoting the degradation of m^6^A-containing circRNAs (Park et al., 2019). To date, no canonical degradation pathway exclusive to circRNAs has been identified. Instead, current evidence suggests that circRNA decay involves multiple pathways, some of which overlap with linear RNA metabolism. The relative contributions of these mechanisms remain unclear, and whether circRNAs are subject to unique, yet undiscovered decay processes is an open question.

## 3 Molecular functions of circRNAs

**FIGURE 3:**
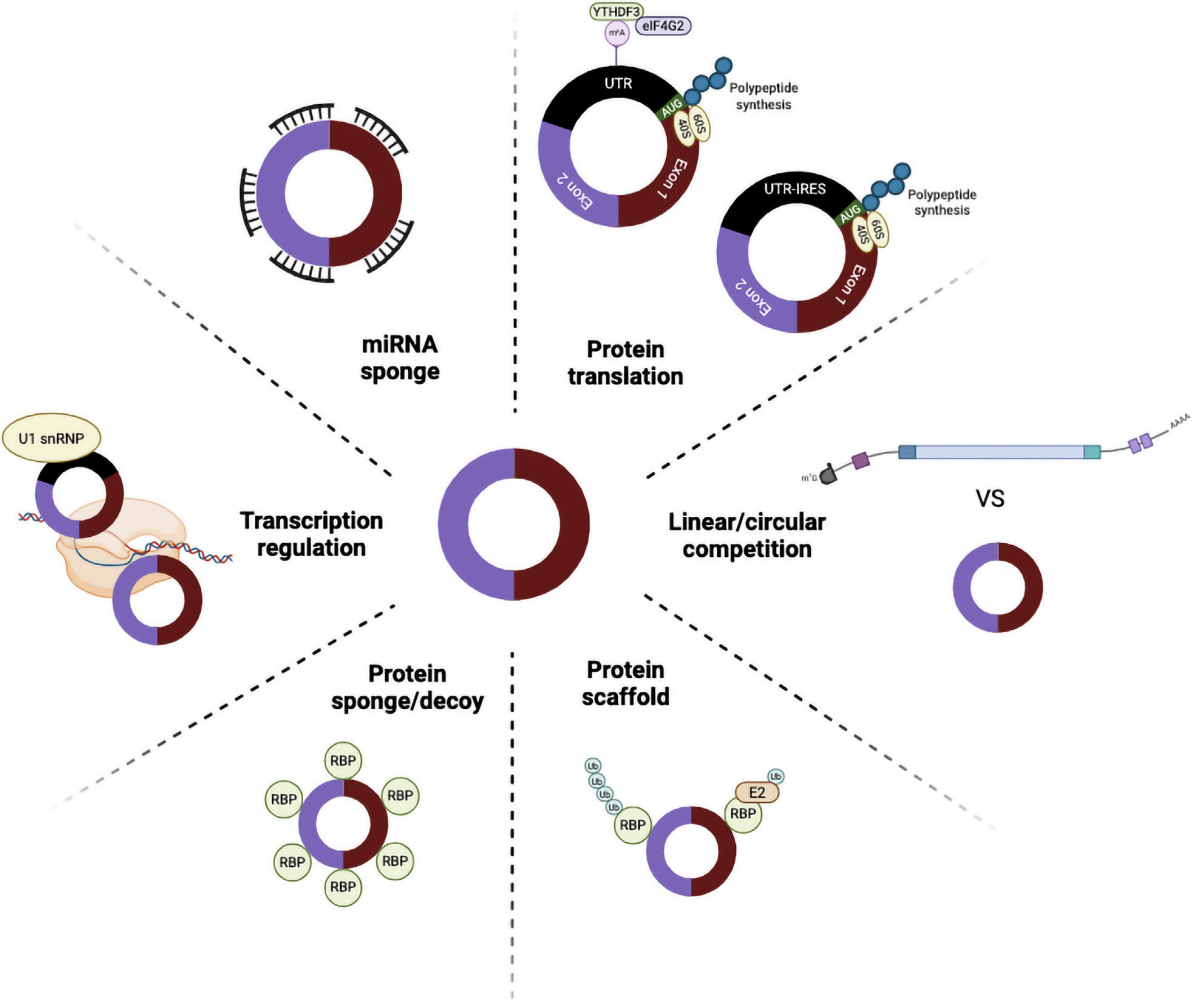
Functional roles of circRNAs in gene regulation and cellular processes. CircRNAs exert diverse biological functions through multiple mechanisms. (i) miRNA sponges: CircRNAs harbour binding sites that sequester miRNAs, preventing them from repressing target mRNAs. (ii) Protein translation: Some circRNAs can be translated into functional peptides either through m^6^A modifications (recognised by YTHDF3 and eIF4G2) or IRES. (iii) Linear/circular competition: CircRNAs compete with their linear mRNA counterparts during splicing, influencing gene expression outcomes. (iv) Transcription regulation: Certain nuclear circRNAs, such as those interacting with U1 snRNP, modulate transcription of their parental genes. (v) Protein sponge/decoy: CircRNAs can bind and sequester RBPs, influencing subcellular localisation, protein activity, and degradation. (vi) Protein scaffold: CircRNAs provide structural platforms for protein–protein interactions, facilitating complex formation, such as ubiquitination cascades. Created in BioRender. Ling, K. (2025) https://BioRender.com/f3mbzja.

CircRNAs are a unique class of RNAs recognised for their diverse molecular functions (Figure 3). They regulate gene expression through multiple mechanisms, primarily by acting as miRNA sponges, thereby modulating miRNA availability and repressing downstream targets. CircRNAs also interact with proteins, influencing their activity, localisation, and stability, and some serve as scaffolds for protein complexes. Moreover, accumulating evidence has revealed that certain circRNAs are translatable, functioning as templates for protein synthesis. Beyond these molecular roles, circRNAs are implicated in broader biological processes, including immune responses, apoptosis, angiogenesis, and hypoxia adaptation. Thousands of distinct circRNAs are expressed in eukaryotic cells, and their functions continue to expand, positioning them as key regulators of cellular physiology and potential therapeutic targets.

### 3.1 CircRNAs as miRNA sponges

One of the most well-characterised functions of circRNAs is their ability to act as miRNA sponges, sequestering miRNAs through complementary binding sequences and thereby reducing their regulatory capacity. Under normal conditions, miRNAs form complexes with AGO2 and bind to their target mRNAs through the conserved 2-8 nucleotide seed region, usually within the 3′UTR, resulting in translational repression or transcript degradation (Bartel, 2018; Shang et al., 2023). CircRNAs containing multiple miRNA response elements can effectively compete with mRNA transcripts, disrupting this post-transcriptional control mechanism. This sponging activity represents a fine-tuned regulatory layer in gene expression networks and has been increasingly implicated in human physiology and disease.

A notable example is circCDR1as, also known as ciRS-7, which contains more than 60 conserved binding sites for *miR-7* (Hansen et al., 2013; Memczak et al., 2013). The circCDR1as exerts strong regulatory control over *miR-7*, thereby influencing numerous downstream targets. Dysregulation of *miR-7* has been associated with abnormal brain and pancreatic development (Latreille et al., 2014; Pollock et al., 2014), as well as with the pathogenesis of Parkinson’s disease (PD) through altered α-synuclein expression (Junn et al., 2009). Beyond the nervous system, circCDR1as has also been implicated in tumour progression, where its overexpression promotes proliferation and metastasis in cancers including nasopharyngeal carcinoma, osteosarcoma, and melanoma (Zhong et al., 2019; Xu B. et al., 2018; Hanniford et al., 2020). Other circRNAs such as circSry, circNRIP1, and circHIPK3 also act as miRNA decoys, sponging *miR-138*, *miR-149*, and *miR-558*, respectively, with significant effects on tumour signalling and progression (Hansen et al., 2013; Zhang et al., 2019; Li et al., 2017).

### 3.2 CircRNAs in translation

Unlike canonical mRNAs, circRNAs lack both a 5′ methylguanosine cap and a 3′ poly(A) tail, which are essential for efficient translation initiation and mRNA stability in eukaryotic cells (Gallie, 1991). Because of their closed-loop structure, circRNAs cannot undergo cap-dependent translation. Instead, they rely entirely on cap-independent mechanisms such as internal ribosome entry sites (IRESs) (Gilbert, 2010). The concept of circRNA translation was first evidenced in the hepatitis delta virus (HDV). Its circRNAincludes an open reading frame (ORF), which directs the synthesis of a hepatitis delta antigen (HDAg) protein (Wang et al., 1986). This finding was later substantiated by studies on the mouse Sry gene, which can generate a circRNA isoform through back-splicing. This circRNA contains an ORF and can produce protein (Capel et al., 1993). Furthermore, high-throughput sequencing and ribosome profiling confirmed that numerous endogenous circRNAs are translated *in vivo*. Well-known examples include circ-ZNF609, circFBXW7, circSHPRH, and circPPP1R12A, all of which encode functional peptides (Abe et al., 2015; AbouHaidar et al., 2014; Wang and Wang, 2014; Gao et al., 2021; Legnini et al., 2017; Pamudurti et al., 2017).

Several mechanisms for cap-independent circRNA translation have since been described. These include IRES-dependent initiation (Gao et al., 2021; Legnini et al., 2017; Pamudurti et al., 2017; Liang et al., 2019), short A/U-rich IRES-like motifs (Mo et al., 2020), m6A-driven translation through m6A-induced ribosome entry sites (MIRES) (Li Y. et al., 2021; Yang et al., 2017), and rolling-circle amplification (RCA)-based translation (Abe et al., 2015). Despite this progress, the exact molecular details of how ribosomal subunits and initiation factors assemble on circRNA templates remain elusive. Evidence suggests that translation is regulated by combinatorial interactions between RBP and components of the eIF4 and eIF3 complexes (Li Y. et al., 2021). Several studies have also demonstrated that circRNAs can bypass canonical cap-dependent translation by utilising IRES, thereby enabling efficient protein production. For instance, circMbl in fruit flies generates peptides that accumulate at synaptic regions under starvation, enhancing synaptic plasticity and stress adaptation (Pamudurti et al., 2017). In humans, circAβ-a from the APP gene translates into an Aβ-containing peptide that aggregates and drives amyloid-β (Aβ) deposition, providing an alternative pathway for Alzheimer’s disease (AD) pathogenesis (Mo et al., 2020).

### 3.3 CircRNAs in transcriptional regulation

Beyond post-transcriptional regulation, circRNAs also influence transcription by interacting with RNA polymerase II or by modulating chromatin-associated proteins. Some circRNAs function as negative regulators, such as metal-responsive element-containing circRNAs. These molecules block the recruitment of gawky (a chromatin-binding RBP) to active loci, thereby impairing copper-stress gene expression (Su et al., 2024). In contrast, certain ciRNAs, such as ci-ankrd52, serve as positive regulators by associating with RNA polymerase II at their parental transcription sites, enhancing gene activity (Zhang et al., 2013). Similarly, EIciRNAs like circEIF3J and circPAIP2 regulate their host gene transcription by forming RNA-RNA interactions with U1 snRNP, which in turn stabilises RNA polymerase II binding (Li Z. et al., 2015).

Other circRNAs exert repressive roles. For example, circHuR directly binds to CNBP, preventing its interaction with the HuR promoter and thereby suppressing HuR transcription, ultimately reducing gastric cancer progression (Yang et al., 2019). Additional transcriptional regulation mechanisms include enhancer-mediated activation, such as sisR-4 in *Drosophila*, which activates an intronic enhancer of the dpn to regulate zygotic transcription (Tay and Pek, 2017). Epigenetic regulation also plays a role, exemplified by FECR1, which recruits TET1 to induce DNA demethylation at the FLI1 promoter, promoting oncogenic transcription and metastasis (Chen N. et al., 2018). Furthermore, cytoplasmic circRNAs can indirectly regulate transcription by sponging miRNAs that normally suppress transcription factors (Chia et al., 2020). Collectively, circRNAs provide a versatile, multilayered control system for transcription, influencing chromatin accessibility, epigenetic states, and the transcriptional machinery.

### 3.4 CircRNAs as protein sponges, decoys and scaffolds

CircRNAs also participate in protein regulation by acting as sponges, scaffolds, and decoys, thereby influencing subcellular localisation, protein activity, and degradation. As molecular sponges, circRNAs directly bind proteins, thereby controlling their activity or localisation. For instance, in colorectal cancer, circYAP1 binds YAP1, blocking its phosphorylation and driving its nuclear import, thereby promoting PD-L1 transcription and allowing tumour cells to evade immune surveillance (Chen et al., 2023). Moreover, in glioblastoma, circ2082 acts as a decoy by binding RBM3, which mislocalises DICER1 to the nucleus and reduces cytoplasmic miRNA abundance (Bronisz et al., 2020). These interactions demonstrate how circRNAs regulate protein function not only by acting as competitive inhibitors but also by serving as decoys that sequester proteins away from their functional sites, thereby reshaping gene expression and immune evasion mechanisms.

In addition to sponging and decoy activities, circRNAs also act as scaffolds and recruiters to coordinate protein-protein interactions and modulate degradation pathways. For example, circAMOTL1 in neonatal cardiac tissue bridges PDK1 and AKT1, enhancing AKT phosphorylation and nuclear translocation, which promotes cardioprotective signalling (Zeng et al., 2017). Similarly, circDNMT1 in breast cancer binds with AUF1 and p53, facilitating their nuclear translocation and contributing to tumour proliferation and senescence escape (Du et al., 2018). CircPABPC1 exemplifies another regulatory dimension by recruiting ITGB1 to the proteasome for ubiquitin-independent degradation in hepatocellular carcinoma (Shi et al., 2021). Conversely, in non-cancerous cells, circFoxo3 functions as an inhibitory scaffold by binding to CDK2 and p21, thereby preventing the formation of the cyclin E/CDK2 complex and blocking the G1/S transition (Du et al., 2016b). Collectively, these examples demonstrate how circRNAs orchestrate complex regulatory networks through scaffolding and recruitment, underscoring their versatile roles in cancer progression, cardiovascular protection, and cell cycle regulation.

### 3.5 CircRNAs and mRNA crosstalk

CircRNAs and their cognate linear mRNAs often exist in a dynamic balance, where the production of one isoform influences the output of the other. One example is circMbl, whose formation is regulated by the splicing factor MBL, encoded by its parental gene. MBL binds to flanking introns of circMbl, driving circularisation. In turn, circMbl binds back to MBL protein, creating a feedback loop that modulates both circular and linear isoform abundance (Ashwal-Fluss et al., 2014). This autoregulatory circuit demonstrates how circRNAs fine-tune splicing decisions and maintain cellular homeostasis. Such interplay highlights the importance of considering both circular and linear RNA outcomes when studying gene regulation.

CircRNAs can also directly influence mRNA stability. Some act by tethering exon junction complexes (EJCs) to the 3′UTR of mRNAs, thereby triggering an NMD-like decay pathway involving PNRC2, UPF1, and UPF2 (Boo et al., 2024). The efficiency of this circRNA-mediated decay depends on the number and positioning of circRNA-mRNA binding sites. Another example is circHOMER1, which exhibits sequence complementarity to the 3′UTR of HOMER1B mRNA. Reduced circHOMER1 expression correlates with increased HOMER1B levels, suggesting a regulatory balance between the two transcripts (Hafez et al., 2022). Moreover, RBPs such as ELAVL4 appear to mediate this interplay by binding to regions near complementary sequences, further stabilising circRNA–mRNA interactions. These findings expand the repertoire of circRNA functions, showing that they not only compete with miRNAs and RBPs but can also directly control transcript abundance and stability.

## 4 CircRNAs in the brain

Analysis of human and mouse neuronal cell lines, along with ENCODE datasets, has consistently shown that circRNAs are more abundant in the mammalian brain than in other tissues (You et al., 2015). For instance, in the human cerebral cortex, 339 circRNAs were identified, of which 141 were cortex-specific (Maass et al., 2017), while large-scale profiling revealed 89,137 circRNAs in the human foetal brain and 65,731 in the adult brain, suggesting a dynamic role for circRNAs in neurodevelopment (Rybak-Wolf et al., 2015). Extending these findings, Dong et al. (Dong et al., 2023) employed laser-capture RNA sequencing (lcRNAseq) to profile the total transcriptome (including non-polyadenylated RNAs) from 190 human post-mortem brains, encompassing dopaminergic neurons, pyramidal neurons, and non-neuronal cells. This approach identified 111,419 candidate circRNAs, of which 11,039 were validated, including 1,526 specific to dopaminergic neurons and 3,308 specific to pyramidal neurons, both enriched in synaptic pathways. Complementing human studies, Xu et al. (Xu K. et al., 2018) identified 17,050 circRNAs in the prefrontal cortex, posterior cingulate cortex, temporal cortex, parietal cortex, occipital cortex, hippocampus, dentate gyrus and cerebellum in the rhesus macaque’s brain. Moreover, analysis of the porcine embryonic brain has identified 4,634 distinct circRNAs (Venø et al., 2015). Among these, 20% of circRNAs were conserved in humans and mice. Together, these findings underscore the diversity, evolutionary conservation, and potential functional significance of circRNAs in brain development, cell-type identity, ageing, and disease.

Three circRNAs, circSATB2, circRIMS1α and circSLC45A4, have been reported to be significantly upregulated during the late fetal stage of human brain development (∼gestational week 24–38), suggesting potential roles in neurogenesis and synaptic plasticity (Chen B. J. et al., 2018). The SATB2 gene encodes a DNA-binding protein that is essential for establishing callosal projection neuron identity and for axon extension across the corpus callosum (Alcamo et al., 2008). Mutations or structural variants affecting SATB2 result in SATB2-associated syndrome, a neurodevelopmental disorder characterised by ID, speech delay, and craniofacial anomalies (Hoed et al., 2021). The RIMS1 encodes a presynaptic active-zone protein that regulates neurotransmitter release and long-term synaptic plasticity. Consistent with this role, knockout of its murine ortholog, Rim1α, leads to severe learning and memory impairments (Schoch et al., 2006; Powell et al., 2004). Moreover, a pathogenic RIMS1 mutation (p.Arg844His) has been associated with autosomal dominant cone–rod dystrophy, and affected individuals exhibit enhanced cognitive performance relative to unaffected relatives (Sisodiya et al., 2007). Functional studies in mice carrying the corresponding Rim1α R655H mutation demonstrated altered regulation of presynaptic calcium channel activity. This dysregulation has been proposed as a potential mechanism linking the variant to both retinal degeneration and enhanced cognitive phenotypes (Miki et al., 2007).

Furthermore, circSLC45A4 is expressed dynamically, peaking at E16.5, a critical period for cortical neurogenesis, and remaining elevated into early P3 in mice (Suenkel et al., 2020). Spatially, circSLC45A4 is broadly expressed across cortical layers at E15.5 and is preferentially enriched in differentiating progenitors and neurons compared with proliferating progenitors at E14.5 (Suenkel et al., 2020). Functionally, *in vivo* knockdown of circSLC45A4 in the developing mouse cortex markedly reduces basal progenitor pools and consequently decreases the number of neurons integrating into the cortical plate (Suenkel et al., 2020). These findings suggest that circSLC45A4 plays a critical role in maintaining the balance between progenitor maintenance and neuronal differentiation. A further example is Cdr1as, also known as ciRS-7 (You et al., 2015). Cdr1as contains over 70 binding sites for *miR-7* and a nearly perfect complementary site for *miR-671* (Piwecka et al., 2017). Cdr1as binding of *miR-7* does not induce degradation due to incomplete pairing, whereas binding of *miR-671* mediates AGO2-dependent cleavage of Cdr1as (Hansen et al., 2011). *In situ* hybridisation studies revealed that Cdr1as is preferentially expressed in excitatory neurons (VGLUT1^+^ and VGLUT2^+^) in the adult mouse brain (Piwecka et al., 2017). Furthermore, single-molecule RNA FISH in primary cortical neurons demonstrated Cdr1as localisation in both soma and neurites, suggesting potential functional roles in regulating post-transcriptional processes across distinct subcellular compartments.

## 5 CircRNAs and Down syndrome

In 2019, the differential expression of circRNAs was analysed in umbilical cord blood from pregnant women carrying a foetus with or without DS using a circular RNA microarray (Sui et al., 2019). The study identified 735 differentially expressed circRNAs, of which 414 were upregulated and 321 were downregulated in the DS group. Among these, RT-qPCR validation was performed on peripheral blood samples from six children with DS and six age- and sex-matched controls (aged 5–12 years). The analysis confirmed significant differences in the expression of six circRNAs. Among them, three were upregulated in DS: hsa_circRNA_103127 (2.64-fold), hsa_circRNA_103112 (2.04-fold), and hsa_circRNA_103135 (4.49-fold). Conversely, three circRNAs were downregulated, including hsa_circRNA_104907 (−4.51-fold), hsa_circRNA_103137 (−2.16), and hsa_circRNA_101116 (−5.07-fold). Bioinformatic analyses suggested that hsa_circRNA_103112 functions as a competing endogenous RNA (ceRNA), sponging miRNAs such as *miR-20b-3p*, *miR-93-3p*, *miR-370-3p*, *miR-520d-3p*, and *miR-651-3p*, implicating it in regulatory networks that may be disrupted in DS. Such interactions exemplify the miRNA “sponge” function of circRNAs, which may contribute to dysregulation of neurodevelopmental and synaptic pathways.

In the foetal hippocampus, Zhao et al. (Zhao et al., 2024) identified 187 differentially expressed circRNAs, including 54 upregulated and 133 downregulated. This study identified one circRNA, hsa_circ_0061697, derived from chromosome 21, which emerged as a central hub in ceRNA networks. It regulates *miR-548b-5p*, *miR-730-5p* and *miR-548i*, thereby reducing their availability to regulate downstream target mRNAs, including SENP3-EIP4, TNC, GART, HELLS, THBS2, B3GALT5, and CLTCL1. Trisomy 21 amplifies this miRNA sponging effect, perturbing PI3K/AKT/mTOR and Wnt signalling, which are critical for neurogenesis, autophagy, and tau phosphorylation. These ceRNA imbalances likely contribute to progressive neuronal defects and cognitive impairment characteristic of DS. Complementing these RNA-seq findings, Wang et al. (Wang et al., 2020) further profiled the foetal hippocampus using high-density circRNA microarrays, identifying 7,078 differentially expressed circRNAs (2,637 upregulated, 4,441 downregulated), predominantly exonic and enriched on chromosome 21. Multiple parental genes produced several circRNAs, including GPC5 (upregulated) and GPR98 (downregulated), linked to synapse-related pathways such as glutamatergic and GABAergic signalling, synaptic vesicle cycling, axon guidance, and long-term potentiation/depression. Disease enrichment analyses connected these circRNAs to intellectual disability and early-onset AD, supporting their relevance to DS cognitive phenotypes. CircRNA expression is disrupted from foetal to adult stages in DS, affecting neuronal development, synaptic plasticity, and cognition. However, longitudinal and stage-resolved studies tracking circRNA expression across developmental stages in DS remain scarce, limiting a precise understanding.

Mechanistic studies highlight circRNA–miRNA–mRNA axes as critical mediators of gene regulation in DS. For example, DS-relevant miRNAs, such as *miR-138-5p*, interact with hub circRNAs, such as hsa_circ_0078328, which sponges *miR-138-5p* to relieve EZH2 repression, thereby influencing neurogenic competence and synaptic plasticity (Wang et al., 2020). As a consequence, the overactivity of factors such as EZH2 leads to abnormal histone methylation, including elevated H3K27me3, and changes in chromatin accessibility (Romero et al., 2024). These epigenetic alterations disrupt the transcriptional programs that govern neural progenitor proliferation, neuronal differentiation, and synapse formation, ultimately contributing to aberrant brain development and cognitive impairment characteristic of DS (Buontempo et al., 2022; Zhang et al., 2023). Moreover, hsa_circ_0061697 modulates the availability of *miR-730-5p* and *miR-548*, affecting downstream targets that converge on the PI3K/AKT/mTOR and Wnt pathways, with consequences for neurodevelopment, autophagy, and tau phosphorylation (Zhao et al., 2024). These alterations impair the clearance of APP/Aβ and phospho-tau, promoting AD-like pathology. Moreover, hub targets include chromatin remodelers and cytoskeletal regulators (e.g., HELLS, TNC, THBS2) that govern neurite stability and synaptic resilience, further implicating circRNA-centred ceRNA networks in disease processes (Zhao et al., 2024). Collectively, these findings demonstrate that trisomy-driven changes in circRNA dosage reshape miRNA availability, disrupt developmental signalling, and alter both structural and functional brain phenotypes, thereby contributing to early-onset AD-like pathology in DS.

The same circRNA-driven regulatory disruptions that predispose to AD-like changes also interfere with neurodevelopmental processes central to cognition, especially molecular mechanisms underlying ID in DS. Evidence shows that aberrant circRNA profiles in umbilical cord blood disrupt post-transcriptional regulation, thereby altering early neurodevelopmental processes (Sui et al., 2019). Such alterations disrupt ceRNA networks, leading to abnormal regulation of genes critical for synaptic plasticity, neuronal differentiation, and neurotransmission (Wang et al., 2020). These disruptions interfere with hippocampal circuitry, a region essential for learning and memory. Consequently, circRNA-mediated dysfunction compromises synaptic connectivity, delays neuronal maturation, and disturbs gene expression balance. The convergence of these molecular abnormalities provides a mechanistic explanation for hippocampal dysfunction, ultimately contributing to the cognitive deficits and intellectual disability characteristic of DS. These studies have shown that circRNAs are crucial regulators of molecular pathology in DS, with most chromosome 21–derived circRNAs being more highly upregulated than those from other chromosomes (Zhao et al., 2024).

Although these studies provide valuable insights into circRNA–miRNA–mRNA regulatory networks in DS, several important issues limit the strength of their conclusions (Table 1). A key concern is the mismatch in tissue sources. A study by Sui et al. (2019) examined umbilical cord and peripheral blood, whereas Zhao et al. (2024) and Wang et al. (2020) focused on foetal hippocampal tissue. This raises uncertainty about whether changes in circulating circRNAs truly mirror brain-specific neurodevelopmental processes. Differences in detection platforms, such as microarray and RNA sequencing, also introduce systematic biases because each approach varies in sensitivity and circRNA annotation coverage. In addition, all three studies rely heavily on computational predictions of miRNA binding and co-expression correlations without experimental validation, leaving the proposed ceRNA interactions largely speculative. None of the studies measure the absolute abundance or subcellular localisation of circRNAs, which are critical factors for determining their capacity to function as miRNA sponges. Statistical and batch-effect issues further complicate data interpretation, increasing the likelihood of artificial network connections. Finally, the biological complexity of DS, characterised by widespread gene dosage effects and cellular heterogeneity, suggests that ceRNA dysregulation is likely only one component of a multifactorial pathogenic process rather than a single unifying mechanism.

**TABLE 1:** Summary of key circRNA–miRNA–mRNA network studies in Down syndrome.

Sample types	Methods	Key findings	Limitation	References
Umbilical cord blood from pregnant women (6 DS fetuses, 6 controls) and peripheral blood from children (6 DS, 6 controls)	CircRNA microarray profiling; RT-qPCR validation; functional annotation of circRNA parental genes using GO and KEGG enrichment analyses	Identified a panel of differentially expressed circRNAs in DS maternal–fetal circulation; proposed that certain circRNAs (e.g., derived from USP25 and RUNX1) could serve as potential non-invasive biomarkers for DS detection	Very small sample size; use of different tissues for discovery and validation (cord vs. peripheral blood); lack of experimental validation of circRNA–miRNA–mRNA interactions; findings are correlative rather than mechanistic	Sui et al. (2019)
Human fetal hippocampal tissue: DS (n = 8) vs. controls (n = 6)	miRNA/mRNA datasets; single-cell RNA-seq (scRNA-seq) annotation for cell-type mapping; Weighted Gene Co-expression Network Analysis (WGCNA); miRNA target prediction (miRanda, miRWalk)	Detected 7,078 differentially expressed circRNAs (2,637 upregulated, 4,441 downregulated); identified 15 hub circRNAs and six gene co-expression modules associated with neuronal development; constructed a core circRNA–miRNA–mRNA ceRNA network potentially regulating neurogenesis and synaptic function in DS hippocampus	Reliance on computational predictions without experimental confirmation; platform bias (microarray vs. RNA-seq); limited biological replicates; heterogeneity across integrated datasets; inferred ceRNA relationships are not functionally tested	Wang et al. (2020)
Human fetal hippocampal samples: DS vs. control (n not specified)	Transcriptome-wide RNA-seq analysis of circRNAs, lncRNAs, miRNAs, and mRNAs; construction of integrated ceRNA and protein–protein interaction networks; functional enrichment (GO, KEGG, GSEA) to identify dysregulated pathways	Revealed extensive ceRNA crosstalk involving lncRNAs, circRNAs, and mRNAs contributing to neuronal differentiation and synaptic dysfunction in DS hippocampus; identified hub regulatory axes potentially linking Chr21 dosage imbalance to downstream molecular pathology	Lack of validation using independent samples or functional assays; unclear sample size and demographic details; reliance on *in silico* network modelling; cannot establish causal relationships between ceRNA dysregulation and neuronal phenotypes	Zhao et al. (2024)

## 6 Integration of circRNA–miRNA–mRNA networks with emerging therapeutic strategies in Down syndrome

Recent transcriptomic analyses have revealed that dysregulated non-coding RNA networks, particularly ceRNA interactions, may play a critical role in the neuropathology of DS. These networks are predicted to influence genes essential for neurodevelopment, synaptic signalling and proteostasis. Building upon this molecular framework, several emerging therapeutic strategies, ranging from molecular and cellular to pharmacological interventions, can be conceptually linked to the modulation of these non-coding RNA regulatory circuits.

Targeting specific non-coding RNA nodes represents a promising molecular therapeutic avenue. When a circRNA functions as a sponge that sequesters miRNAs repressing key neurodevelopmental mRNAs, therapeutic interventions could aim to restore regulatory balance. For example, antisense oligonucleotides or small interfering RNAs directed against the unique back-splice junctions of pathogenic circRNAs could diminish their expression, thereby releasing miRNAs to resume normal repression of downstream targets (Løvendorf et al., 2023). Conversely, the use of miRNA mimics or inhibitors (antagomirs) could adjust miRNA activity levels and normalise dysregulated gene expression. Such sequence-specific modulation of ceRNA components complements existing gene-targeted approaches by offering a fine-tuned means of correcting post-transcriptional dysregulation. Nevertheless, as circRNA studies remain largely predictive and correlative, these proposed interventions will require rigorous functional validation to establish causality before translation into clinical practice.

Stem-cell-based therapies may also intersect with ceRNA regulatory dynamics. Mesenchymal stem cells (MSCs) and induced pluripotent stem cell (iPSC)-derived neural cells are known to exert neuroprotective effects (Laterza et al., 2013; Shahryari et al., 2025). These effects are mediated in part through the secretion of extracellular vesicles (EVs) enriched in miRNAs, circRNAs, and other regulatory molecules (Upadhya et al., 2020; Volarevic et al., 2025). These EVs can modify gene expression and inflammatory responses in recipient cells, suggesting that stem cell therapies could beneficially remodel ceRNA networks within the DS hippocampal microenvironment. Although the circRNA studies did not directly demonstrate such paracrine transfer or establish causality, integrating ceRNA profiling into stem-cell research could clarify whether these therapies achieve part of their neurorestorative effects through modulation of non-coding RNA communication.

Pharmacological approaches that target neuroinflammation and intracellular signalling cascades may exert secondary effects on ceRNA network homeostasis. Neuroinflammatory mediators such as IL-1β and TNF-α are known to influence transcriptional and post-transcriptional gene regulation, including non-coding RNA expression (Jiang et al., 2022). Consequently, anti-inflammatory or neuroprotective treatments might indirectly normalise pathological circRNA or miRNA profiles. For instance, if a pro-inflammatory milieu upregulates a circRNA that sequesters a miRNA critical for synaptic gene translation, attenuating inflammation could restore appropriate ceRNA balance. This mechanistic link underscores the value of coupling molecular profiling with pharmacological interventions to assess whether clinical improvements correlate with shifts in ncRNA network activity (Lorenzon et al., 2023).

Moreover, an emerging and innovative direction involves the prediction and mapping of circRNA, miRNA, and mRNA networks as therapeutic strategies in their own right. In this approach, ceRNA network models derived from transcriptomic and bioinformatic analyses are used to identify new molecular targets or to guide therapeutic repurposing. For instance, the study by Zamanian Azodi et al. (2021) used network centrality analysis to identify hub genes, including RHOA, FGF2, FYN, and CD44, that may contribute to DS pathology beyond chromosome 21. Targeting these network “bottleneck” nodes could potentially influence broader regulatory pathways. Similarly, Pecze and Szabo (2021) reported extensive mitochondrial and bioenergetic gene expression disturbances in DS brains, suggesting that ceRNA networks involving mitochondrial transcripts could serve as intervention targets. Integrating these insights with the circRNA–miRNA–mRNA axes provides a conceptual framework for precision therapeutics. In such a model, predicted ceRNA hubs could serve as direct targets for antisense oligonucleotides or miRNA mimics. At the same time, network nodes connecting ceRNA modules to inflammatory or synaptic pathways could be addressed through pharmacological or extracellular vesicle–based approaches. Ultimately, this predictive framework transforms ceRNA dysregulation from a descriptive biomarker concept into a structured therapeutic roadmap, enabling the design of interventions guided by network architecture rather than by single-gene focus.

In summary, converging data indicate that ceRNA network dysfunction constitutes a mechanistic layer linking gene-dosage imbalance to neuronal pathology in DS. Integrating ncRNA-directed molecular therapies, stem-cell-based modulation, pharmacological anti-inflammatory strategies, and predictive network mapping may together offer a coherent, multi-level framework for future therapeutic design. However, translating these concepts into clinical practice will require extensive functional validation, optimisation of delivery systems, and longitudinal assessment of safety and efficacy across developmental stages.

## 7 Future direction

In summary, mounting evidence suggests that DS-associated circRNAs, particularly those originating from chromosome 21, actively contribute to neuropathological features such as impaired synaptic plasticity, altered immune signalling, and early-onset Alzheimer’s-like pathology. These observations underscore that circRNAs are dynamic regulators rather than mere byproducts of splicing. Despite this progress, the understanding of circRNA function in DS remains limited. Most studies are descriptive, focusing on differential expression, and the variability of circRNA profiles across tissues, developmental stages, and cell types complicates efforts to establish causal links. Furthermore, the interactions of circRNAs with other regulatory layers, including epigenetics, proteomics, and metabolomics, remain largely unexplored. Integrating multi-omics approaches with advanced bioinformatics and machine learning could provide a systems-level understanding of circRNA networks and their functional relevance.

Recent evidence has illuminated how circRNAs orchestrate key neurodevelopmental pathways, offering mechanistic insights directly relevant to DS. High-abundance circRNAs are significantly enriched in biological categories such as synaptic signalling, neuronal projection development, and maintenance of synaptic structure, which are tightly coupled to fundamental developmental events, including rapid neural growth, neural tube closure, and accelerated neuronal differentiation (Yang et al., 2025). RNA-sequencing studies of DS foetal hippocampus and umbilical cord blood have identified thousands of differentially expressed circRNAs, including hsa_circRNA_103112 and hsa_circ_0061697, that regulate miRNAs involved in synaptic pathways, neurotransmission, and neuronal differentiation. These circRNA–miRNA–mRNA regulatory networks perturb major developmental signalling cascades such as PI3K/AKT/mTOR and Wnt, ultimately contributing to impaired neurogenesis, disrupted glutamatergic–GABAergic balance, and early-onset Alzheimer’s-like pathology in DS.

Complementing these transcriptomic findings, emerging research identifies m^6^A RNA methylation as a pivotal epigenetic regulatory mechanism that maintains transcriptional and translational equilibrium during central nervous system development (Yang et al., 2021). Because circRNAs can also undergo m^6^A modification, disturbances in this pathway (common in DS due to oxidative stress, mitochondrial dysfunction, and chromosomal imbalance) may alter circRNA stability, subcellular localisation, and capacity to act as miRNA sponges. Such m^6^A-circRNA dysregulation likely amplifies the downstream epigenetic and transcriptional disruptions already driven by trisomy 21. In this context, circRNA-mediated modulation of factors such as EZH2, which influences histone H3K27me3 deposition and chromatin remodelling, further links RNA-based and epigenetic regulation to the cognitive and structural brain abnormalities characteristic of DS.

Building on these insights, future studies should aim to clarify the molecular mechanisms by which circRNAs contribute to neural dysfunction and cognitive impairment in DS. A key priority is the comprehensive identification of chromosome 21–derived circRNAs involved in synaptic regulation, particularly those influencing the balance between excitatory and inhibitory neurotransmission. Elucidating how these circRNAs affect synaptic signalling and plasticity will help uncover the molecular pathways underlying altered neuronal connectivity and cognitive deficits. Further investigation into the specific mechanistic roles of individual circRNAs, including their interactions with target miRNAs and mRNAs, is essential for understanding their contributions to abnormal gene expression and neural development. Longitudinal studies examining circRNA expression dynamics across developmental stages will provide valuable insights into their temporal regulation and their roles in disease progression. Special attention should be directed toward exosome-associated circRNAs, which may mediate intercellular communication and reflect systemic molecular changes in DS. Additionally, comparative analyses of circRNA expression patterns between brain and peripheral tissues, such as blood or plasma, are needed to determine whether peripheral samples can serve as reliable biomarkers of brain-specific molecular alterations. Collectively, advancing functional validation, integrating multi-omics datasets, and pursuing translational studies will be essential for uncovering the precise roles of circRNAs in DS pathogenesis. Such efforts will deepen our understanding of how molecular dysregulation contributes to cognitive and neural abnormalities in Down syndrome. They will also open new avenues for developing innovative diagnostic and therapeutic strategies targeting circRNA-mediated pathways.
